# Supplementary material for: Simultaneously Predicting the Pharmacokinetics of CES1-Metabolized Drugs and Their Metabolites Using Physiologically Based Pharmacokinetic Model in Cirrhosis Subjects
Source: Pharmaceutics. 2024 Feb 5;16(2):234. doi: 10.3390/pharmaceutics16020234 (PMC10893190; doi:10.3390/pharmaceutics16020234)
Supplement: Supplementary file 1 [file pharmaceutics-16-00234-s001.zip › pharmaceutics-2796812-supplementary.pdf]

The specific formulation of the semi-PBPK model is shown below.
It was assumed that no absorption and metabolism of drugs occurs in stomach. The amount ($A_0$) in stomach is controlled by the constant of gastric emptying rate ($K_{t,0}$), i,e

$$\frac{dA_0}{dt} = -K_{t0} \times A_0 \quad (S1)$$

Small intestine is divided into duodenum, jejunum and ileum, which is further divided into the gut lumen and the gut wall. Drug amount ($A_i$) in the i^th gut lumen is illustrated by

$$\frac{dA_i}{dt} = K_{t,i-1} \times A_{i-1} - K_{ti} \times A_i - k_{a,i} \times A_i \quad (S2)$$

Where $K_{t,i}$ represents the constant of intestinal transit rate. $k_{a,i}$ represents the absorption rate constant from the gut lumen to the gut wall, which may be calculated using equation,

$$k_{ai} = \frac{2 \times P_{eff,A-B}}{r_i} \quad (S3)$$

Where $r_i$ is the intestinal radius. $P_{eff\ A\text{-}B}$ is effective permeability coefficient ($P_{eff}$) from gut lumen to gut wall. The $P_{eff}$ ($\times 10^{-4}$) values were estimated using the in vitro apparent permeability coefficient of drugs ($P_{app} \times 10^{-6}$) in Caco-2 cells based the equation[1]:

$$LogP_{eff} = 0.4926 \times LogP_{app,Caco-2} - 0.1454 \quad (S4)$$

The drug concentration in the $i^{th}$ gut wall ($C_{GWi}$) is expressed as follows:

$$\frac{V_{GWi} \times dC_{GWi}}{dt} = k_{a,i} \times A_i + Q_{GWi} \times C_{sys} - Q_{GWi} \times C_{GWi} \times R_b/K_{G:P} \quad (S5)$$

Where $Q_{GWi}$ and $K_{G:P}$ represent the blood flow rate in $i^{th}$ gut wall and ratio of drug concentration in intestinal wall to plasma, respectively. $C_{GWi}$ and $V_{GWi}$ represent separately drug concentration in the $i^{th}$ intestinal and wall volume of the $i^{th}$ gut wall. $C_{sys}$ represent drug concentrations in the systemic compartment. $R_b$ is the ratio of drug concentrations in blood to plasma.
The drug enters the liver through the portal vein and the concentration in the portal vein ($C_{PV}$) is:

$$\frac{V_{PV} \times dC_{PV}}{dt} = \Sigma Q_{GWi} \times C_{GWi} \times R_b/K_{G:P} - Q_{PV} \times C_{PV} \quad (S6)$$

$Q_{PV}$ and $C_{PV}$ represent portal vein blood flow rate and volume of portal vein, respectively.
It was assumed that metabolism of CES1-mediated drugs mainly occurs in liver. Drug concentration ($C_L$) in liver is illustrated by

$$\frac{V_L \times dC_L}{dt} = Q_{PV} \times C_{PV} + Q_{LA} \times C_{sys} - Q_L \times C_L \times R_b/K_{L:P} - CL_{int} \times f_{u,b} \times C_L \times R_b/K_{L:P} \quad (S7)$$

Where $Q_{LA}$ and $Q_L$ represent the hepatic artery blood flow rate to the liver and hepatic blood flow to the systemic compartment, respectively. $V_L$ and $K_{L:P}$ represent the volume of liver and ratio of drug concentration in liver to plasma, respectively. $CL_{int}$ and $f_{u,b}$ represent intrinsic clearance in the liver and free fraction of drug in blood, respectively. $f_{u,b}$ is generated from the fraction unbound in plasma ($f_{u,p}$), i.e

$$f_{u,b} = \frac{f_{u,p}}{R_b} \quad (S8)$$

$CL_{int}$ can be estimated using in vitro enzyme kinetics from human hepatic microsomes.

$$CL_{int} = \Sigma \frac{V_{max,i}}{K_{m,i} + f_{u,b} \times \frac{A_L \times R_b}{V_L \times K_{L;P}}} \approx \Sigma \frac{V_{max,i}}{K_{m,i}} \quad (S9)$$

Where $V_{max,i}$ and $K_{m,i}$ represent the maximum velocity and Michaelis-Menten constant in vitro enzyme kinetic experiments, respectively.
Hepatic clearance ($CL_L$) of a drug may be derived from total CL ($CL_T$) and renal CL ($CL_K$), i.e

$$CL_L = CL_T - CL_K \quad (S10)$$

Thus, The $CL_{int}$ is also recalculated by hepatic blood clearance ($CL_{L,b}$) using equation

$$CL_{L,b} = \frac{Q_L \times f_{u,b} \times CL_{int}}{Q_L + f_{u,b} \times CL_{int}} \quad (S11)$$

The CL values by clinic are often plasma clearance of drug ($CL_P$), which may be transferred to blood clearance ($CL_b$) using equation 12.

$$CL_b = \frac{CL_p}{1 - Hct + R_b \times Hct} \quad (S12)$$

Where Hct is hematocrit, 0.43 in healthy subjects[2].

Some metabolites of some drugs are also eliminated via bile. Amount of metabolites ($A_{L,m}$) in liver is illustrated by equation 13.

$$\frac{V_L \times dC_{L,m}}{dt} = Q_{PV} \times C_{PV,m} + Q_{LA} \times C_{sys,m} + CL_{int,CSE1} \times f_{u,b} \times C_L \times R_b / K_{L:p} - Q_L \times C_{L,m} \times R_{b,m} / K_{L:P,m} - (CL_{int,m} + CL_{int,b,m}) \times f_{u,b,m} \times C_{L,m} \times R_{b,m} / K_{L:P,m} \quad (S13)$$

Where $CL_{int,b,m}$ and $CL_{int,\,m}$ are intrinsic bile clearance and intrinsic metabolic clearance, respectively. If metabolism of the metabolite did not occur in the body, the $CL_{int,b,m}$ may be recalculated from $CL_K$ using equations 10 and 11.

Kidney is involved in elimination of drugs, especially their metabolites. Amount of drugs in kidney is illustrated by equation

$$\frac{V_K \times dC_K}{dt} = Q_K \times C_{sys} - (Q_K + f_{u,b} \times CL_{int,K}) \times C_K \times R_b / K_{K,P} \quad (S14)$$

Where $Q_K$ and $V_K$ represent kidney blood flow and volume of the kidney, respectively. $CL_{int,K}$ and $K_{K,P}$ represent intrinsic clearance in kidney and tissue-to-plasma concentration ratio in the kidney, respectively. $CL_{int,K}$ was also estimated from $CL_K$ using equation 11.

Disposition of drugs in the systemic compartment is illustrated using one-compartment, two-compartment model or three-compartment model.

For one-compartment model

Drug concentration ($C_{sys}$) in systemic compartment

$$\frac{V_{sys} \times dC_{sys}}{dt} = Q_L \times C_L \times R_b / K_{L:p} + Q_K \times C_K \times R_b / K_{K:p} - (Q_{LA} + Q_K) \times C_{sys} - \sum Q_{GWi} \times C_{sys} \quad (S15)$$

For two-compartment model

Drug concentration ($C_{sys}$) in systemic compartment.

$$\frac{V_{sys} \times dC_{sys}}{dt} = Q_L \times C_L \times R_b / K_{L:P} + Q_K \times C_K \times R_b / K_{K:P} + k_{21} \times A_p - k_{12} \times V_{sys} \times C_{sys} - (Q_K + Q_{LA}) \times C_{sys} - \sum Q_{GWi} \times C_{sys} \quad (S16)$$

$$\frac{dA_P}{dt} = k_{12} \times V_{sys} \times C_{sys} - k_{21} \times A_P \quad (S17)$$

For three-compartment model

Drug concentration ($C_{sys}$) in systemic compartment.

$$\frac{V_{sys} \times dC_{sys}}{dt} = Q_L \times C_L \times R_b / K_{L:P} + Q_K \times C_K \times R_b / K_{K:P} + k_{21} \times A_{p1} - k_{12} \times V_{sys} \times C_{sys} + k_{31} \times A_{p2} - k_{13} \times V_{sys} \times C_{sys} - (Q_K + Q_{LA}) \times C_{sys} - \sum Q_{GWi} \times C_{sys} \quad (S18)$$

$$\frac{dA_{P1}}{dt} = k_{12} \times V_{sys} \times C_{sys} - k_{21} \times A_{P1} \quad (S19)$$

$$\frac{dA_{P2}}{dt} = k_{13} \times V_{sys} \times C_{sys} - k_{31} \times A_{P2} \quad (S20)$$

Where $V_{sys}$ represents the apparent distribution volume in systemic compartment. $A_P$ and $A_{P1}$ are the amount of drug in two peripheral compartments. $k_{12}$, $k_{21}$, $k_{13}$ and $k_{31}$ represent the transfer rates between the systemic compartment and peripheral compartment.

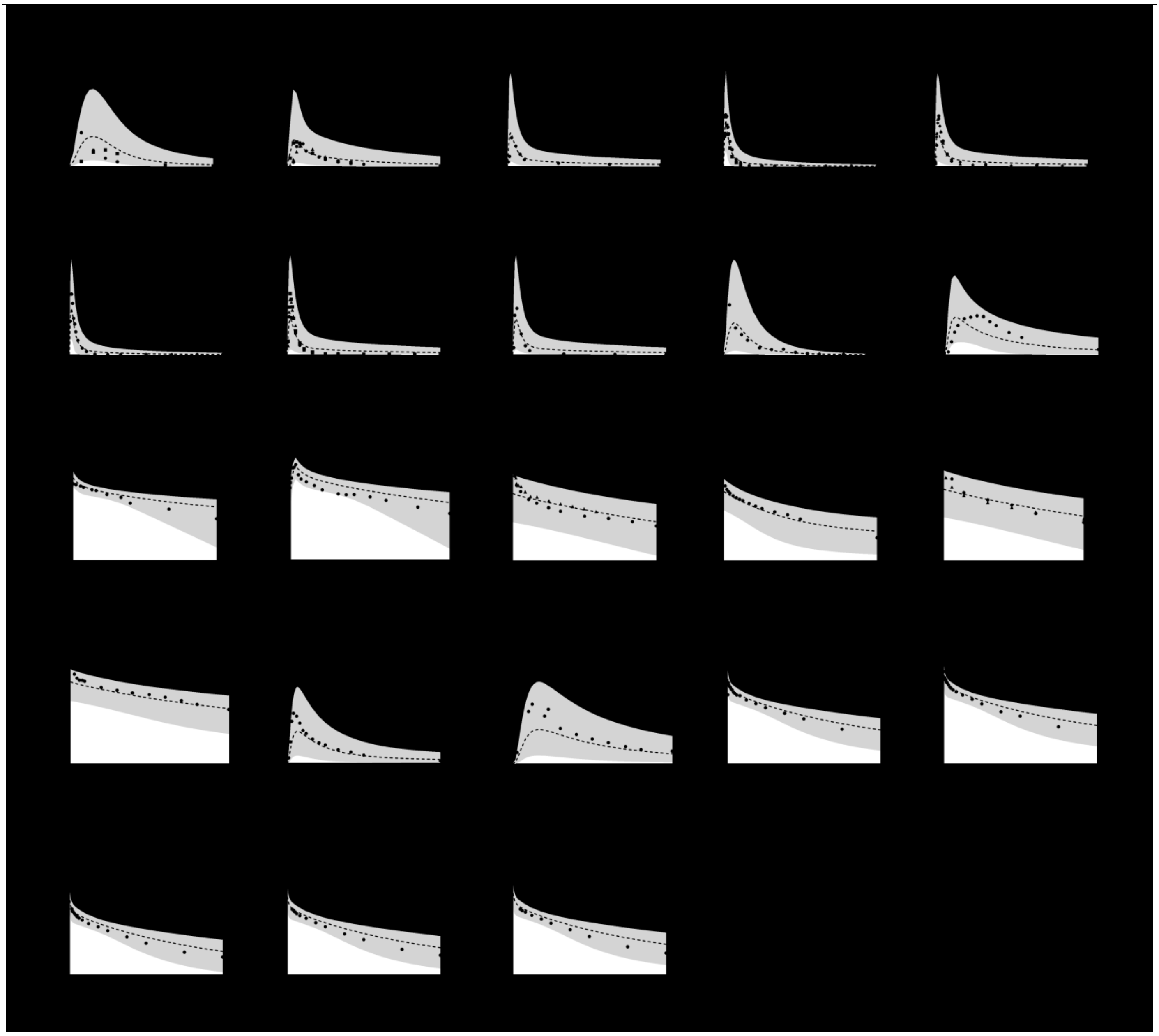

**Figure S1.** The observed (points) and predicted (lines) plasma concentrations of the tested CES1 substrates and their active metabolites following intravenous or oral administration to healthy subjects. Benazepril **(A)**[3,4] and benazeprilat **(B)**[3-5] following oral 10 mg benazepril hydrochloride; cilazapril **(D**, **F)**[6,7] and cilazaprilat **(C**, **E**, **G**, **H)**[6-9] following oral 1.25, 2.5, 5, 10 mg cilazapril; oseltamivir **(I)**[10] and oseltamivir carboxylate **(J)**[10] following oral 150 mg oseltamivir phosphate; flumazenil following intravenous 10 mg/1 min **(K)**[11] and 10mg/10min **(L)**[12]; pethidine following intravenous 50 mg/1min **(M)**[13], 25mg/1min **(N)**[14], 0.8mg/kg,1min **(O)**[15] and 0.8mg/kg,5min **(P)**[16]; pethidine hydrochloride and oral 25 mg **(Q)**[14], 0.8mg/kg **(R)**[16] pethidine hydrochloride; remimazolam following intravenous 0.05 **(S)**[17], 0.075 **(T)**[17], 0.2 **(U)**[17], 0.3 **(V)**[17], 0.4 **(W)**[17] mg/kg remimazolam besylate. Shaded areas indicate the 5th and 95th percentiles of simulations derived from 1000 virtual individuals. The dashed lines indicate the mean of the simulated profiles.

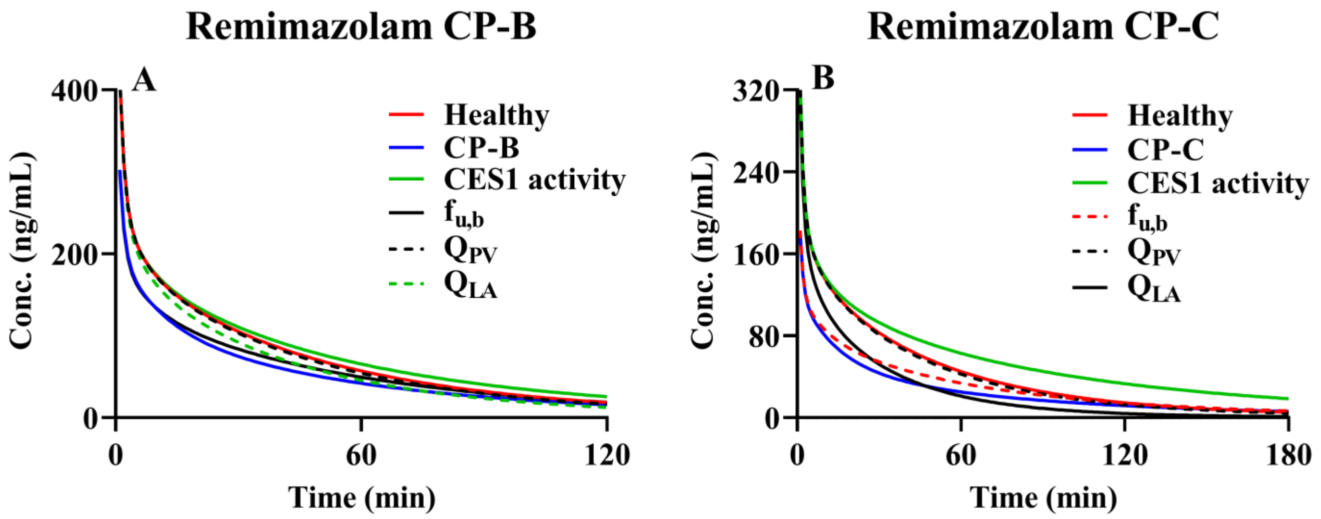


**Figure S2.** Contributions of LC-induced alterations in $f_{u,b}$, CES1 activity, $Q_{LA}$ and $Q_{PV}$ to plasma concentration of remimazolam following 10.4mg (CP−B, A) and 8.2mg (CP−C, B) administration to healthy human and LC patients.

References:


1. Gertz, M.; Harrison, A.; Houston, J.B.; Galetin, A. Prediction of human intestinal first-pass metabolism of 25 CYP3A substrates from in vitro clearance and permeability data. *Drug Metab Dispos* **2010**, *38*, 1147-1158, doi:10.1124/dmd.110.032649.
2. Edginton, A.N.; Willmann, S. Physiology-based simulations of a pathological condition: prediction of pharmacokinetics in patients with liver cirrhosis. *Clin Pharmacokinet* **2008**, *47*, 743-752, doi:10.2165/00003088-200847110-00005.
3. Kaiser, G.; Ackermann, R.; Brechbuhler, S.; Dieterle, W. Pharmacokinetics of the angiotensin converting enzyme inhibitor benazepril.HCl (CGS 14 824 A) in healthy volunteers after single and repeated administration. *Biopharm Drug Dispos* **1989**, *10*, 365-376, doi:10.1002/bdd.2510100404.
4. Schweizer, C.; Kaiser, G.; Dieterle, W.; Mann, J. Pharmacokinetics and pharmacodynamics of benazepril hydrochloride in patients with major proteinuria. *Eur J Clin Pharmacol* **1993**, *44*, 463-466, doi:10.1007/BF00315544.
5. Macdonald, N.J.; Sioufi, A.; Howie, C.A.; Wade, J.R.; Elliott, H.L. The effects of age on the pharmacokinetics and pharmacodynamics of single oral doses of benazepril and enalapril. *Br J Clin Pharmacol* **1993**, *36*, 205-209, doi:10.1111/j.1365-2125.1993.tb04218.x.
6. Massarella, J.; DeFeo, T.; Lin, A.; Limjuco, R.; Brown, A. The pharmacokinetics and dose proportionality of cilazapril. *Br J Clin Pharmacol* **1989**, *27 Suppl 2*, 199S-204S, doi:10.1111/j.1365-2125.1989.tb03482.x.
7. Williams, P.E.; Brown, A.N.; Rajaguru, S.; Francis, R.J.; Bell, A.J.; Dewland, P.M. Pharmacokinetics of cilazapril during repeated oral dosing in healthy young volunteers. *Eur J Drug Metab Pharmacokinet* **1990**, *15*, 63-67, doi:10.1007/BF03190129.
8. Francis, R.J.; Brown, A.N.; Kler, L.; Fasanella d'Amore, T.; Nussberger, J.; Waeber, B.; Brunner, H.R. Pharmacokinetics of the converting enzyme inhibitor cilazapril in normal volunteers and the relationship to enzyme inhibition: development of a mathematical model. *J Cardiovasc Pharmacol* **1987**, *9*, 32-38.
9. Massarella, J.W.; DeFeo, T.M.; Brown, A.N.; Lin, A.; Wills, R.J. The influence of food on the pharmacokinetics and ACE inhibition of cilazapril. *Br J Clin Pharmacol* **1989**, *27 Suppl 2*, 205S-209S, doi:10.1111/j.1365-2125.1989.tb03483.x.
10. Jittamala, P.; Pukrittayakamee, S.; Tarning, J.; Lindegardh, N.; Hanpithakpong, W.; Taylor, W.R.; Lawpoolsri, S.; Charunwattana, P.; Panapipat, S.; White, N.J.; et al. Pharmacokinetics of orally administered oseltamivir in healthy obese and nonobese Thai subjects. *Antimicrob Agents Chemother* **2014**, *58*, 1615-1621, doi:10.1128/AAC.01786-13.
11. Amrein, R.; Hetzel, W. Pharmacology of Dormicum (Midazolam) and Anexate (Flumazenil). *Acta Anaesth Scand* **1990**, *34*, 6-15, doi:DOI 10.1111/j.1399-6576.1990.tb03174.x.
12. Breimer, L.T.; Hennis, P.J.; Burm, A.G.; Danhof, M.; Bovill, J.G.; Spierdijk, J.; Vletter, A.A. Pharmacokinetics and EEG effects of flumazenil in volunteers. *Clin Pharmacokinet* **1991**, *20*, 491-496, doi:10.2165/00003088-199120060-00005.
13. Mather, L.E.; Tucker, G.T.; Pflug, A.E.; Lindop, M.J.; Wilkerson, C. Meperidine kinetics in man. Intravenous injection in surgical patients and volunteers. *Clin Pharmacol Ther* **1975**, *17*, 21-30, doi:10.1002/cpt197517121.
14. Verbeeck, R.K.; Branch, R.A.; Wilkinson, G.R. Meperidine disposition in man: influence of urinary pH and route of administration. *Clin Pharmacol Ther* **1981**, *30*, 619-628, doi:10.1038/clpt.1981.213.
15. Klotz, U.; McHorse, T.S.; Wilkinson, G.R.; Schenker, S. The effect of cirrhosis on the disposition and elimination of meperidine in man. *Clin Pharmacol Ther* **1974**, *16*, 667-675, doi:10.1002/cpt1974164667.
16. Neal, E.A.; Meffin, P.J.; Gregory, P.B.; Blaschke, T.F. Enhanced Bioavailability and Decreased Clearance of Analgesics in Patients with Cirrhosis. *Gastroenterology* **1979**, *77*, 96-102, doi:10.1016/s0016-5085(79)80017-7.
17. Sheng, X.Y.; Liang, Y.; Yang, X.Y.; Li, L.E.; Ye, X.; Zhao, X.; Cui, Y.M. Safety, pharmacokinetic and pharmacodynamic properties of single ascending dose and continuous infusion of remimazolam besylate in healthy Chinese volunteers. *Eur J Clin Pharmacol* **2020**, *76*, 383-391, doi:10.1007/s00228-019-02800-3.
